# Regulation of mouse *Scgb3a1 *gene expression by NF-Y and association of CpG methylation with its tissue-specific expression

**DOI:** 10.1186/1471-2199-9-5

**Published:** 2008-01-14

**Authors:** Takeshi Tomita, Shioko Kimura

**Affiliations:** 1 Laboratory of Metabolism, National Cancer Institute, National Institutes of Health, Bethesda, Maryland 20892, USA

## Abstract

**Background:**

Secretoglobin (SCGB) 3A1 is a secretory protein of small molecular weight with tumor suppressor function. It is highly expressed in lung and trachea in both human and mouse, with additional tissues expressing the protein that differ depending on the species. However, little is known about the function and transcriptional regulation of this gene in normal mouse tissues.

**Results:**

By reporter gene transfection and gel mobility shift analyses, we demonstrated that expression of the mouse *Scgb3a1 *gene is regulated by a PU-box binding protein and a ubiquitous transcription factor NF-Y that respectively binds to the PU-boxes located at -99 to -105 bp and -158 to -164 bp, and the "CCAAT" binding sites located at -425 to -429 bp and -498 to -502 bp from the transcription start site of the gene. However, the effect of PU-box binding protein on transcriptional activation is minimal as compared to NF-Y, suggesting that NF-Y is a more critical transcription factor for mouse *Scgb3a1 *gene transcription. Despite that NF-Y is a ubiquitous factor, *Scgb3a1 *is highly expressed only in mouse lung and mtCC cells that are derived from SV40 transformed mouse Clara cells, but not in ten other mouse tissues/cells examined. Gene methylation analysis revealed that within 600 bp of the *Scgb3a1 *gene promoter region, there are nine CpG methylation sites present, of which two CpGs closest to the transcription start site of the gene are unmethylated in the tissues/cells expressing SCGB3A1.

**Conclusion:**

A ubiquitous transcription factor NF-Y binds to and activates expression of the mouse *Scgb3a1 *gene and tissue-specific expression of the gene is associated with CpG methylation of the promoter.

## Background

Secretoglobin (SCGB) 3A1, also called high in normal-1 (HIN-1) [1] or uteroglobin-related protein 2 (UGRP2) [2], is a member of the SCGB gene superfamily that consists of small secretory proteins [3]. HIN-1 was originally identified as a tumor suppressor gene because its expression was silenced by methylation in the majority of human breast carcinomas [1]. UGRP2 was independently identified as a homologous gene to UGRP1 (also called SCGB3A2) that is a downstream target for the homeodomain transcription factor NKX2-1, also called TITF1, TTF1, NKX2.1, or T/EBP [2]. Mouse SCGB3A1 and SCGB3A2 share 33% amino acid sequence identity [2].

In humans, SCGB3A1 is highly expressed in the trachea, lung, salivary gland, prostate, esophagus, duodenum and mammary gland [1,2,4], whereas in mouse, it is primarily expressed in the trachea and lung, and weakly expressed in the heart, stomach, and small intestine [2,4,5]. Methylation patterns of the human *SCGB3A1 *gene promoter have been extensively studied and a correlation of methylation and loss of SCGB3A1 expression and malignant phenotypes is well established in many human cancers including breast, prostate, lung, and pancreatic carcinomas [6-8]. The AKT signaling pathway is responsible for the SCGB3A1's tumor suppressor function as characterized by inhibition of cell growth, cell migration and invasion [9]. In this connection, it was shown that EGF and TGFγ increase SCGB3A1 expression through activation of the ERK-MAPK and phosphoinositide-3 kinase-AKT pathways [10]. Further, the expression of SCGB3A1 is restricted to terminally differentiated airway epithelial cells and is up-regulated during retinoic acid induced differentiation of bronchial epithelial cells, suggesting that SCGB3A1 may be involved in the acquisition or maintenance of the terminally differentiated epithelial phenotype [5].

Under interleukin (IL)-4 and IL-13 stimulation, SCGB3A1 expression is up-regulated through binding of STAT6 to the STAT binding element located in -201 to -209 bp of the mouse *Scgb3a1 *gene promoter [11], suggesting that SCGB3A1 may also play a role in inflammation. In fact, a recent report demonstrated that SCGB3A2 (UGRP1), a homologous gene to SCGB3A1, suppresses allergic airway inflammation when a mouse model for allergic airway inflammation is subjected to intranasal administration of recombinant adenovirus expressing SCGB3A2 [12]. STAT6 is the only transcription factor downstream of IL-4 and IL-13 signaling thus far demonstrated that directly binds to the *Scgb3a1 *promoter and regulates expression of the gene [11]. It is not known what transcription factors are involved in constitutive expression of the mouse *Scgb3a1*.

In this study, we demonstrate that a ubiquitous transcription factor NF-Y is an important transcription factor for controlling expression of the mouse *Scgb3a1 *gene through its binding to the responsive "CCAAT" element located at -425 to -429, and -498 to -502 bp upstream of the *Scgb3a1 *gene. The methylation pattern of the mouse *Scgb3a1 *gene is examined and the association of the two closest CpGs to the transcription start site in tissue-specific expression of the mouse *Scgb3a1 *gene is discussed.

## Results

### Analysis of the mouse *Scgb3a1 *gene promoter

**Figure 1:**
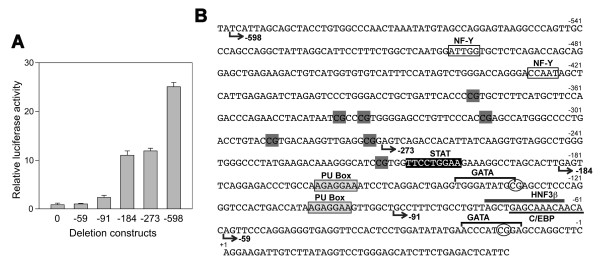
**Reporter analysis of the mouse *Scgb3a1 *gene**. (**A**) Transient transfection of serial deletion luciferase reporter constructs into mtCC cells. Relative luciferase activity is shown as the mean ± SD based on the activity of pGL4 (shown as construct 0) as 1 from three independent experiments, each carried out in duplicate. (**B**) Mouse *Scgb3a1 *promoter sequence of -600 bp to the transcription start site. Start points of the deletion constructs, -59, -91, -184, -273, and -598 are shown. NF-Y binding 'CCAAT" sites are boxed. PU-boxes are shown in shaded boxes. A STAT binding site previously described for IL-4/13-induced increase of *Scgb3a1 *gene expression is shown in black box with white letters. GATA, HNF3β, and C/EBP binding sites identified by low stringency TF search analysis are indicated by over-bracket, overlined and underlined, respectively. CpG methylation sites are indicated in a gray box for complete methylation, and circled for unmethylated sites (see also Fig. 7 and Table 1).

In an attempt to understand the mechanism of mouse *Scgb3a1 *gene expression, we first compared the promoter sequence of the mouse *Scgb3a1 *gene with the human *SCGB3A1 *gene. Interestingly, no significant homology was found by BLAST analysis when 2-kbp mouse *Scgb3a1 *gene promoter sequence was subjected to analysis, suggesting that the regulation of human and mouse *Scgb3a1 *genes may be quite different. Six mouse *Scgb3a1 *gene promoter-luciferase reporter constructs (pGL4, -59, -91, -184, -273, and -598) were then prepared and subjected to transient transfection analysis using transformed mouse Clara cells (mtCC) that are derived from tumor tissues of lungs obtained from transgenic mice expressing the simian virus 40 large T antigen gene under control of uteroglobin/Clara cell secretory protein promoter [13]. The reporter activity slightly increased with construct -91 (Fig. 1A). An approximately ten-fold enhancement of promoter activity as compared to a control construct pGL4 was obtained with the -184 construct, and the extent of activity remained at similar levels up to -273 bp of the promoter sequence. A further robust up-regulation (~25-fold) of the promoter activity as compared to control was obtained with construct -598, suggesting that transcriptional response elements may be present between -91 and -184 bp, and -273 and -598 bp of the mouse *Scgb3a1 *gene promoter. Possible binding sites for transcription factors were analyzed using the TF search [14], TRANSFAC [15] and the reference of [16]. The analysis revealed the presence of two PU boxes (5'-AGAGGAA-3') between -91 and -184 bp and two NF-Y binding sites (5'-CCAAT-3') between -273 and -598 bp that may be responsible for up-regulation of *Scgb3a1 *gene expression (Fig. 1B). The PU-box is a binding site for the ETS family of transcription factors that participate in an array of cellular activities in development, differentiation and tumorigenesis, and is a main regulator of the immune system [17,18]. NF-Y is a ubiquitous heterotrimeric transcription factor, composed of NF-YA, NF-YB, and NF-YC, all of which are necessary for DNA binding, and recognizes "CCAAT" penta-nucleotide element [19-22]. A STAT binding site (-201 to -209 bp) that we previously characterized [11] for IL-4/13-induced increase of SCGB3A1 expression did not seem to be involved in the constitutive expression of SCGB3A1. Further TF search analysis with low stringency revealed a possible binding site for GATA (-11 to -20 bp and -132 to -141 bp), C/EBP (-61 to -72 bp) and HNF3β (-64 to -76 bp), transcription factors known to be involved in lung-specific gene expression [23]; the latter two might contribute a slight increase of the reporter activity found with the -91 construct.

### NF-Y is involved in the mouse *Scgb3a1 *gene transcription

**Figure 2:**
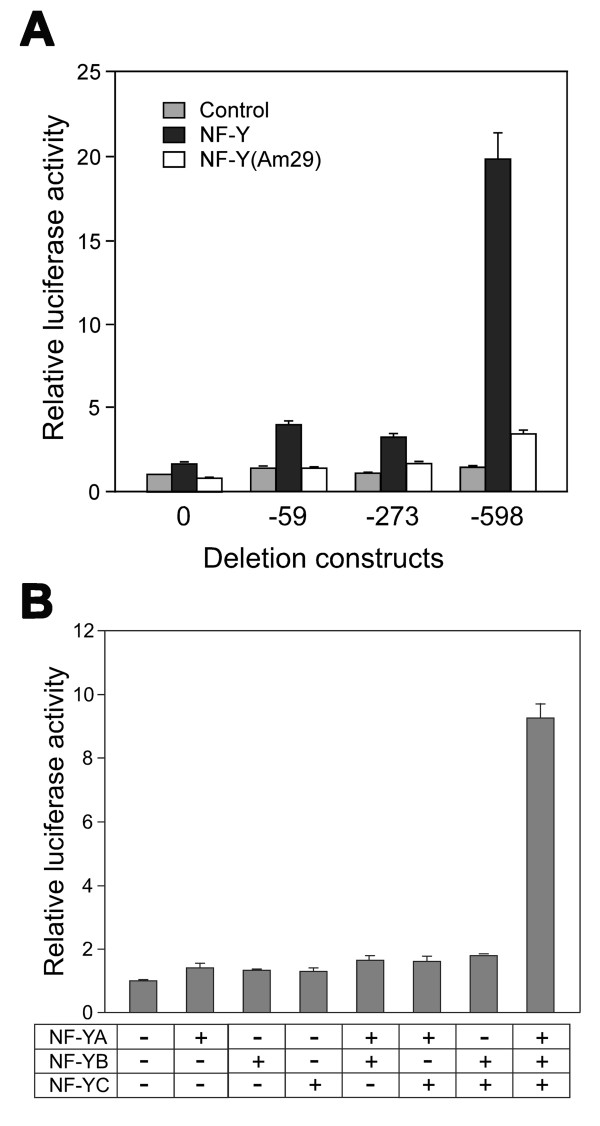
**NF-Y comprised of all three subunits is required for mouse *Scgb3a1 *gene transcription**. (**A**) Transient transfection of serial deletion luciferase reporter constructs into COS-1 cells in the absence (control), or presence of NF-Y or its dominant negative form NFY(Am29). Relative luciferase activity is shown as the mean ± SD based on the activity of pGL4 (shown as construct 0) as 1 from three independent experiments, each carried out in duplicate. (**B**) -598 reporter construct was co-transfected with various combination of NF-Y subunits into COS-1 cells. Relative luciferase activity is shown as the mean ± SD based on the activity of construct -598 without any NF-Y subunits as 1 from three independent experiments, each carried out in duplicate.

The involvement of NF-Y in mouse *Scgb3a1 *gene transcription was first examined by co-transfecting pGL4 control vector, -59, -273, and -598 *Scgb3a1*-luciferase constructs with expression plasmids for three NF-Y subunits into COS-1 cells that do not express SCGB3A1 (Fig. 2A). Only construct -598 has putative NF-Y binding sites. Luciferase activity increased approximately two-fold with the constructs -59 and -273. Further inclusion of upstream sequence up to -598 bp markedly increased luciferase activity to almost twenty-fold as compared to control, confirming the involvement of NF-Y in mouse *Scgb3a1 *gene transcription. In order to examine whether all three NF-Y subunits are required for expression of SCGB3A1, co-transfecting analysis was carried out using single or a various combinations of NF-Y subunits (Fig. 2B). The results clearly demonstrate that all three NF-Y subunits are required for *Scgb3a1 *gene transcription as previously described [21]. Further, replacing the NF-YA construct with NF-YAm29, a dominant negative form of NF-YA that lacks the DNA binding activity due to displacement of three critical amino acids in the middle of the DNA binding domain but retaining the ability to form the NF-Y heterotrimer [24], NF-Y up-regulation of -598 luciferase activity was reduced to close to basal levels, suggesting that the NF-YA subunit is critical for transcriptional activation (Fig. 2A). These results demonstrate that NF-Y may activate mouse *Scgb3a1 *transcription by binding to the NF-Y binding site(s) located between -273 and -598 bp of the *Scgb3a1 *gene promoter.

**Figure 3:**
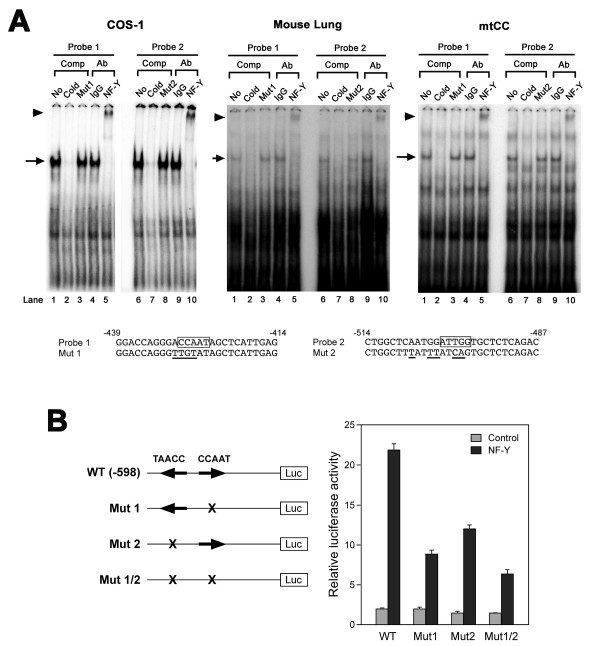
**Analysis of NF-Y binding sites**. (**A**) EMSA analysis of NF-Y binding sites. Proximal (Probe 1: -425 to -429 bp, lane 1–5) and distal (Probe 2: -498 to -502 bp, lane 6–10) "CCAAT" binding sites in the mouse *Scgb3a1 *gene promoter were incubated with nuclear extracts prepared from COS-1 cells over-expressing NF-Y, mouse lung and mtCC cells in the presence of no competitor (lane 1, 6), 200-fold excess of own cold oligonucleotide as a competitor (lane 2, 7), 200-fold excess of mutant 1 or 2 as a competitor (lane 3 or 8, respectively), rabbit IgG as a control antibody (lane 4, 9), and polyclonal anti-NF-Y (anti-NF-YA and NF-YB combined) (lane 5, 10). NF-Y-DNA complex is indicated by an arrow, and NF-Y supershifted band is indicated by an arrowhead. Sequences for probe 1 and 2, and their mutants are shown at the bottom. These mutations were designed based on [37] and modification was added until complete abolishment of NF-Y binding was obtained. "CCAAT" sites are boxed and the mutated bases are underlined. (**B**) Transfection analysis of mutant constructs. COS-1 cells were co-transfected with -598 wild-type or a mutant construct (Mut 1, Mut 2) in the presence or absence of NF-Y expression plasmid. An arrow indicates the direction of the "CCAAT" sequence. Relative luciferase activity is shown as the mean ± SD based on the activity of wild-type construct -598 in the absence of NF-Y as 1 from three independent experiments, each carried out in duplicate.

In the -273 to -598 bp region, two "CCAAT" sequences are present at -425 to -429 bp and -498 to -502 bp (reverse direction) from the transcription start site (Fig. 1B). In order to determine which putative binding sites are involved in binding of NF-Y that is responsible for the increase of *Scgb3a1 *gene promoter activity, electrophoretic gel mobility shift analysis (EMSA) was carried out using double strand oligonucleotides containing proximal (-425 to -429 bp) or distal (-498 to -502 bp) "CCAAT" box as a probe and nuclear extracts prepared from COS-1 cells over-expressing NF-Y, mouse lungs, or mtCC cells (Fig. 3A). In all three nuclear extracts, the same pattern of specific protein-DNA shifted band was obtained, although intensity varied, for both probes 1 and 2 (lane 1 and 6); the specific band was competed out by a 200-fold excess of cold probe (lane 2 and 7), but not by an oligonucleotide having a mutation at the "CCAAT" site (lane 3 and 8). Further, polyclonal anti-NF-Y antibody specifically super-shifted the NF-Y-DNA complex with both probes (lane 5 and 10), which was not seen with IgG (lane 4 and 9) or anti- C/EBP antibody (data not shown) as a negative control. These results demonstrate that NF-Y binds to the two "CCAAT" binding elements located at -425 to -429 and -498 to -502 bp in the *Scgb3a1 *gene promoter.

In order to determine which NF-Y binding element is responsible for *Scgb3a1 *gene transactivation, mutant reporter plasmids were constructed by introducing mutations singly or doubly into two of the "CCAAT" binding sites in the -598 construct (Fig. 3B). Mutations introduced were the same as those used for EMSA (see Fig. 3A). When the proximal "CCAAT" binding site was mutated (Mut 1), luciferase reporter activity with co-transfection of NF-Y expression plasmid was reduced to approximately one-third as compared to wild-type, whereas Mut 2 having the distal "CCAAT" site mutated, displayed about one-half the luciferase activity of wild-type. With both mutations together, luciferase activity was further reduced to approximately one-fourth of that of wild type. These results suggest that the proximal NF-Y binding site may be slightly more responsible, however, both NF-Y binding sites are required for *Scgb3a1 *gene activation.

### NF-Y is a dominant transcription factor over PU-box binding protein in the mouse *Scgb3a1 *gene promoter activity

**Figure 4:**
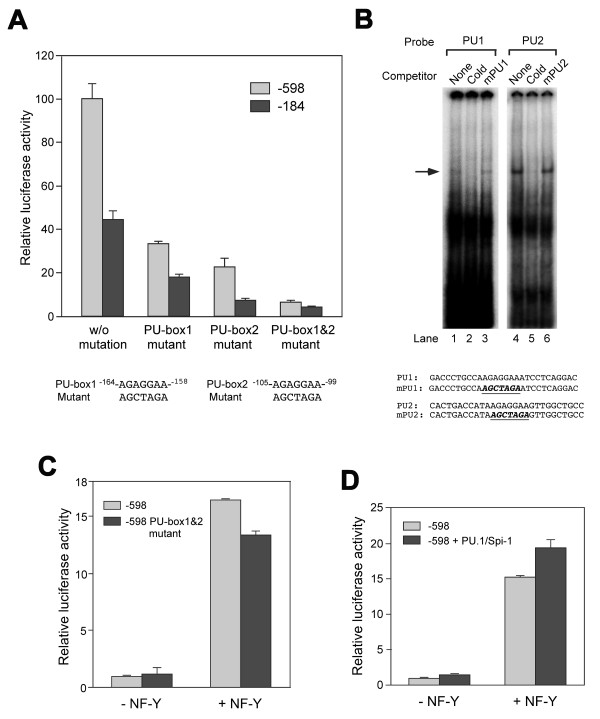
**Analysis of PU-boxes**. (**A**) Transfection analysis of the constructs -184 and -598, and their respective PU-box 1 and 2 mutants into mtCC cells. Mutations introduced into PU-box 1 and 2 are shown at the bottom. Relative luciferase activity is shown as the mean ± SD based on the activity of construct -598 without (w/o) mutation as 100 from three independent experiments, each carried out in duplicate. (**B**) EMSA analysis of PU-box 1 (PU1) and 2 (PU2). EMSA was carried out using oligonucleotide containing PU1 or 2 binding site as a probe (shown at the bottom) and nuclear extracts prepared from mtCC cells. Mutation was introduced into each binding site (mPU1, mPU2 in bold italics with underline) and was used as a competitor. Both PU1 and PU2 bound the same protein as shown by an arrow. (**C**) Transfection analysis of -598, and -598 PU-box 1 and 2 mutants into COS-1 cells with and without NF-Y. (**D**) Cotransfection analysis of construct -598 and PU.1/Spi-1 expression plasmid into COS-1 cells in the presence and absence of NF-Y. Relative luciferase activity is shown as the mean ± SD based on the activity of the construct -598 without NF-Y as 1 from three independent experiments, each carried out in duplicate.

The role of two PU-boxes (located at -99 to -105 bp and -158 to -164 bp from the transcription start site, named PU-box 1 and PU-box 2, respectively) in the mouse *Scgb3a1 *gene promoter activity was examined by transfection analysis of mutant constructs in mtCC cells. Mutations were introduced into the PU-boxes singly or doubly in the -184 and -598 constructs, the latter containing two NF-Y binding sites (Fig. 4A). In both -184 and -598 constructs, PU-box 1 and 2 individual mutants showed markedly reduced reporter activities at the similar extent in relative to their parent constructs, in which PU-box 2 appeared to be more responsible for the promoter activity. The activity was further reduced to a basal level by both mutations together. EMSA using mtCC nuclear extracts demonstrated that oligonucleotide containing PU-box 1 or 2 produced the same specific DNA-protein band, which was competed out by a cold probe, but not a mutated probe (mPU1 or mPU2) (Fig. 4B). These results suggest that both PU-boxes 1 and 2 may be required for transcription of the mouse *Scgb3a1 *gene.

To determine which transcription factor between NF-Y and PU-box binding protein is more critical for the regulation of mouse *Scgb3a1 *gene, co-transfection experiments were carried out in COS-1 cells using the construct -598 and -598 PU-box1&2 double mutant with and without NF-Y. While both constructs had very little reporter activity without NF-Y (also see Fig. 4A), the promoter activity was markedly increased by NF-Y over-expression regardless of the presence of PU-box double mutation (Fig. 4C). The importance of NF-Y in mouse *Scgb3a1 *gene transcription was further demonstrated by co-transfection into COS-1 cells of the -598 construct and an expression plasmid for PU.1 (purine rich box-1)/Spi-1 (SFFV (spleen focusforming virus) proviral integration site-1), one of the PU box binding ETS family transcription factors [25,26], although we do not know what ETS family member of transcription factor is involved in mouse *Scgb3a1 *gene transcription. In this case, *Scgb3a1 *promoter activity was slightly increased by the addition of PU.1/Spi-1 expression plasmid both in the absence and presence of NF-Y, however, again the effect of NF-Y on the promoter activity was far more robust than the PU-box binding transcription factor (Fig. 4D). These results suggest that NF-Y may be a dominant transcription factor over PU-box binding protein for mouse *Scgb3a1 *gene transcription. We therefore focused on NF-Y in further studies.

### Analysis of NF-Y binding to its specific binding sites in the mouse *Scgb3a1 *gene promoter

**Figure 5:**
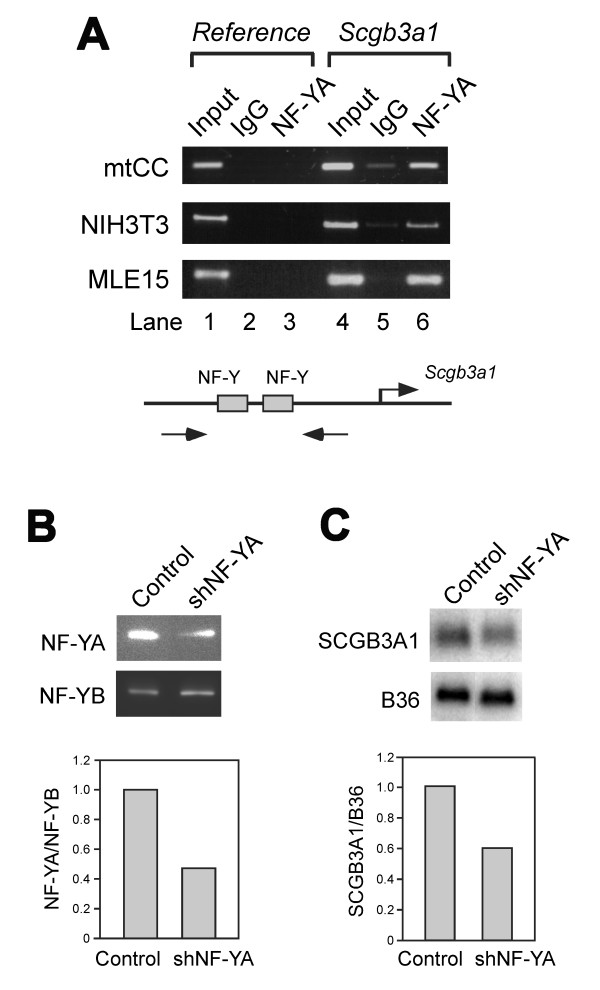
**NF-Y controls expression of *Scgb3a1 *gene**. (**A**) ChIP analysis of mtCC, NIH3T3, and MLE15 cells for endogenous NF-Y binding. Antibodies used were NF-YA and IgG as a control. A sequence derived from the exon II of *Scgb3a2*, a homologous gene to *Scgb3a1 *was used as a reference gene. Illustration of the PCR strategy is shown at the bottom. Experiments were repeated twice and the same results were obtained. (**B**) Western blot analysis of NF-YA protein level after NF-YA shRNA transfection into mtCC cells. Experiments were repeated twice and the same results were obtained. Representative western blot result is shown with the quantitated bar graph at lower panel. (**C**) Northern blot analysis of SCGB3A1 mRNA level after NF-YA shRNA transfection into mtCC cells. Experiments were repeated twice and the same results were obtained. Representative northern blot result is shown with the quantitated bar graph at lower panel.

In order to further demonstrate that NF-Y is a critical transcription factor that binds to the promoter of *Scgb3a1 *gene and activates expression of the gene, chromatin immunoprecipitation (ChIP) experiments were carried out using mtCC cells that constitutively express SCGB3A1 [10,11], and NIH3T3 and mouse immortalized respiratory cell-derived MLE15 cells [27] that do not express SCGB3A1. ChIP analysis demonstrated that NF-Y binds to *Scgb3a1 *gene promoter in all three cell lines examined regardless of SCGB3A1 expression (Fig. 5A and Table 1). Further, when NF-YA shRNA was transfected to mtCC cells, the amount of NF-YA protein was reduced to approximately a half as compared to NF-YB subunit, whose level stayed the same after NF-YA shRNA transfection (Fig. 5B). At the same time, SCGB3A1 mRNA levels were reduced to approximately 60% (Fig. 5C). These results together with the results described above, demonstrate that NF-Y is necessary and perhaps sufficient for *Scgb3a1 *gene expression through binding to the "CCAAT" binding sites located in the mouse *Scgb3a1 *gene promoter.

**Table 1:** Correlation between *Scgb3a1 *promoter methylation and gene expression in various mouse tissues/cells

Mouse tissues/cells	Proximal 2 CpGs Methylation	SCGB3A1 expression
Brain	Yes	No
Heart	Yes	Very low
Kidney	Yes	No
Liver	Yes	No
Lung	Methylated + Unmethylated	High
Skeletal Muscle	Yes	No
Spleen	Yes	No
Testis	Yes	No
mtCC	Unmethylated	High
MLE 15	Yes	No
Hepa 1	Yes	No
NIH3T3	Yes	No

### CpG methylation plays a role in the regulation of mouse *Scgb3a1 *gene

**Figure 6:**
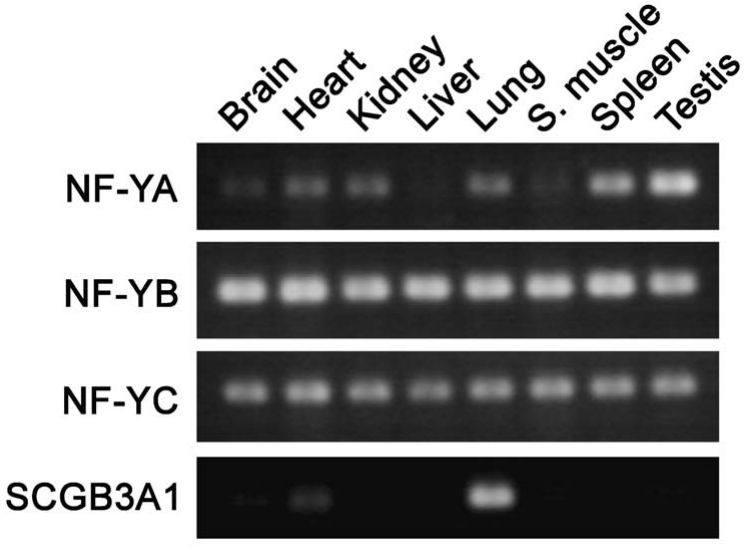
**RT-PCR analysis for the expression of NF-YA, NF-YB, NF-YC, and SCGB3A1**. RNAs isolated from various organs of adult mouse were subjected to RT-PCR. S. Muscle; skeletal muscle. Note that high SCGB3A1 expression is found only in lung.

While NF-Y is a ubiquitous factor [20-22] and binds to the mouse *Scgb3a1 *gene promoter, the expression in mouse is mainly found in the trachea and lung [2,4,5]. In order to understand the mechanism for tissue-specific expression of SCG3A1, RT-PCR was first carried out to determine the levels of expression of three NF-Y subunits, NF-YA, NF-YB, and NF-YC, and SCGB3A1 in various mouse tissues including brain, heart, kidney, liver, lung, skeletal muscle, spleen and testis (Fig. 6). NF-YB and NF-YC were expressed in all tissues examined at similar levels, whereas expression of the NF-YA subunit varied depending on tissue/cell types as previously described [21]: expression in the brain, liver and skeletal muscle was very low or barely detected as compared with other tissues. Interestingly, SCGB3A1 was highly expressed only in the lung with very weak expression detected in the heart. These results confirmed the ubiquitous pattern of NF-Y expression, which does not correlate with SCGB3A1 expression.

**Figure 7:**
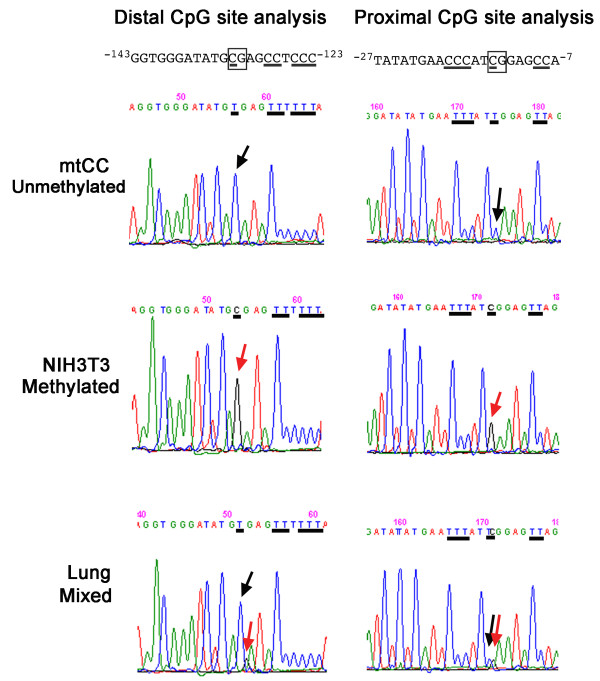
**DNA methylation analysis**. Distal and proximal CpG sites of the mouse *Scgb3a1 *gene promoter were subjected to direct DNA sequencing after bisulfite treatment and PCR amplification of DNAs isolated from mtCC, NIH3T3 and mouse lungs. Sequence containing CpG site is shown at the top, and CpG site is boxed. Cytosine residues are underlined. If these cytosine residues are unmethylated, they appear as thymine after bisulfite treatment (upper panel; mtCC unmethylated, shown by a black arrow). The cytosine residue that is methylated stays as cytosine even after bisulfite treatment (middle panel; NIH3T3 methylated, shown by a red arrow). Lung DNA shows both T and C residues due to a mixture of cell types of methylated and unmethylated (bottom panel; Lung mixed, shown by a black and red arrow).

In order to understand the tissue-specific expression of SCGB3A1, the methylation pattern in the mouse *Scgb3a1 *promoter was examined. It is well known that DNA methylation plays an important role in tissue-specific gene expression [28]. To this end, genomic DNA was subjected to bisulfite modification, and the DNA fragment spanning -600 bp upstream through +90 bp in the intron 1 of the mouse *Scgb3a1 *gene was PCR amplified, followed by a direct DNA sequencing of the PCR fragment to determine the site of CpG methylation in the promoter and around the transcription start site of the gene. This modification converts non-methylated cytosine to uracil but does not affect 5-methyl cytosine. In the region between +90 to -600 bp of the mouse *Scgb3a1 *gene, nine CpG methylation sites were found, all of which were located between NF-Y binding sites and the transcription start site (Fig. 1B). Of nine, seven methylation sites located at the most 5' were always methylated regardless of tissues/cell lines examined, whereas two methylation sites closest to the transcription start site were partially or totally unmethylated in lung or mtCC cells, respectively (Fig. 7 and Table 1). When 28 individual clones obtained from PCR products of bisulfite modified lung DNAs were subjected to sequencing, 10 were found to have the two CpG sites closest to the transcription start site methylated while 18 were unmethylated. These results suggest that lung DNAs consist of a mixture of methylated and unmethylated CpGs. Note that direct sequencing of DNAs from lung, mtCC and NIH3T3 cells without bisulfite modification did not demonstrate any mutations anywhere within +90 to -600-bp region. Interestingly, lung and mtCC were the only tissues/cells that exhibited high level of SCGB3A1 expression. Together with the results obtained by ChIP analysis, in which all mtCC, NIH3T3, and MLE15 cells have bound NF-Y at its specific binding sites of the mouse *Scgb3a1 *promoter regardless of SCGB3A1 expression, and that NF-Y is ubiquitously expressed in all tissues/cells examined, the bisulfite sequencing results demonstrate that two methylation sites closest to the transcription start site in the *Scgb3a1 *promoter may be responsible for tissue-specific expression of SCGB3A1.

**Figure 8:**
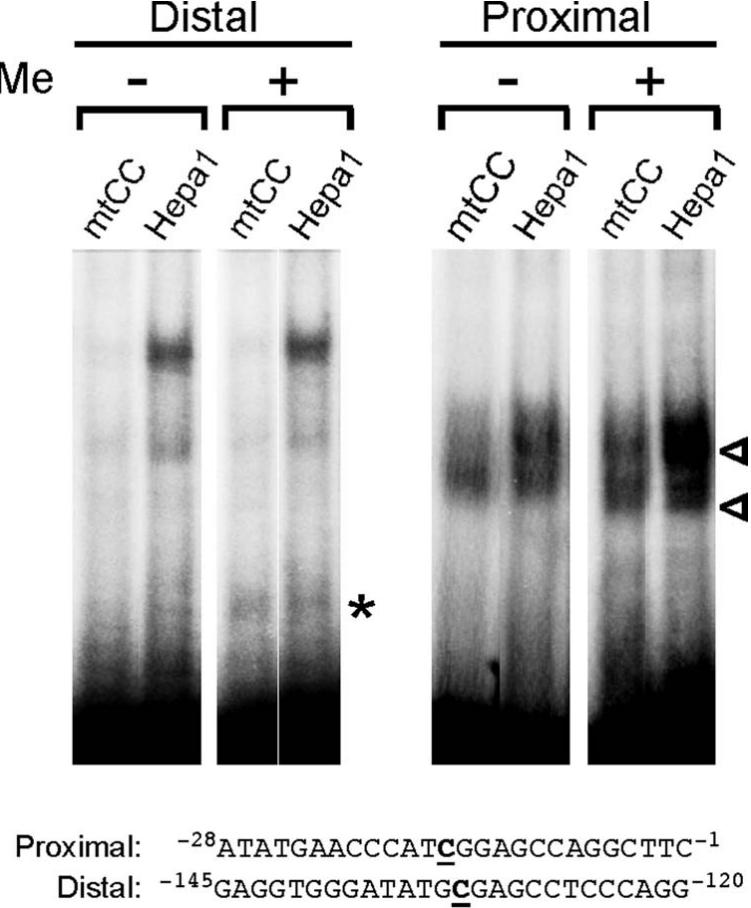
**EMSA with methylated CpG oligunucleotides**. EMSA was carried out using nuclear extracts prepared from mtCC or Hepa 1 cells and oligonucleotides containing proximal or distal CpG site methylated (Me +) or unmethylated (Me -). Oligonucleotide sequences are shown at the bottom with methylated C underlined in boldface. Shifted bands only seen with methylated CpG oligonucleotide are shown by open arrowhead for proximal or asterisk for distal CpG region.

Lastly, in order to examine whether CpG methylation affects binding of DNA-binding proteins, we performed EMSA using an oligonucleotide having the proximal or distal CpG methylated as compared with unmethylated oligonucleotide (Fig. 8). When CpG was methylated, an additional band(s) was observed for both oligonucleotides flanking proximal or distal CpG using nuclear extracts prepared from mtCC and mouse hepatoma-derived Hepa1 cells. Since the shifted band is at different position between two oligonucleotides, they may represent different DNA-binding proteins. These results suggest that CpG methylation affects the binding of DNA-binding proteins.

## Discussion

We report here for the first time that a ubiquitous transcription factor NF-Y is important for regulation of the mouse *Scgb3a1 *gene, while DNA methylation appears to be associated with tissue-specific expression of the gene. NF-Y recognizes a "CCAAT" penta-nucleotide that can be found at every 500 bp in random genomic DNAs based on mathematical calculation. With additional high stringency, in which the "CCAAT" box is expanded to a 9-nucleotide element as (G/A)(G/A)CCAAT(C/G)(A/G)(G/C) [20], this sequence appears every 16 kbp in frequency. A "CCAAT" box is found in the vicinity of promoter region (between -60 and -100 bp of the major start site in both orientations) with significant high frequency (30%), suggesting that NF-Y may play a pivotal role in controlling expression of many genes [29]. In the current study, CCAAT boxes were located further upstream (-425 to -429 bp and -498 to -502 bp), away from the vicinity of *Scgb3a1 *gene transcription start site.

Transfection analysis in COS-1 cells revealed that the -59 and -273 constructs increased reporter activity two-fold in an NF-Y dependent manner (see Fig. 2A). However, no "CCAAT" binding sequence was found between -59 bp and the transcription start site, nor did EMSA reveal any NF-Y binding in this region (data not shown). While an exact "CCAAT" sequence is required for NF-Y binding, "CCACT" sequence was reported to similarly function as the NF-Y binding site in regulation of Oncoprotein18 gene [30]. A low stringency survey revealed the presence of two "CCACT" sequences in the *Scgb3a1 *gene promoter at -44 to -48 bp and -113 to -117 bp, however, neither bound NF-Y as determined by EMSA (data not shown). Further, when NFYAm29, a dominant negative form of NF-YA was co-transfected with the constructs -598, an approximately two-fold higher luciferase activity as compared to control remained even though a heterotrimer with NF-Yam29 cannot bind DNA (Fig. 2A) [24]. Moreover, mutation of the two NF-Y binding sites did not completely abolish NF-Y activation of the -598 construct luciferase activity (Fig. 3B). The exact reason for these phenomena is not known. However, it has been established that NF-Y interacts with TATA-binding protein (TBP) [31], TFIID [31], and coactivators such as p300 [32] and P/CAF [33] that modify gene expression. The interaction of NF-Y with these factors could stabilize basic transcription machinery and/or its association with DNA, which in turn results in a slight increase in *Scgb3a1 *reporter activity with co-transfection of constructs -598 and NF-YAm29, and/or the constructs -59/-273 and NF-Y expression plasmid. Note that in these reporter assays, three expression constructs, NF-YA, NF-YB, and NF-YC were simultaneously co-transfected along with a reporter construct. The fact that only cells that have taken up all four plasmids can produce a complete form of NF-Y that can activate the promoter suggests that the 20-fold increase of *Scgb3a1 *promoter activity obtained in this co-transfection assay system may represent only a fraction of naturally occurring NF-Y regulated *Scgb3a1 *expression.

The importance of NF-Y, in particular the NF-YA subunit in mouse *Scgb3a1 *gene expression was demonstrated by reporter activities using a various combination of NF-Y subunits and reduced SCGB3A1 mRNA expression by NF-YA shRNA. In the latter, both NF-YA protein and SCGB3A1 mRNA levels were suppressed at similar levels of approximately 50–60% of the control. This incomplete suppression may be due to the fact that NF-Y is a ubiquitous transcription factor and regulates many genes including housekeeping genes and those involved in cell cycle control, that account for approximately 20% of all genes [20,22,34]. Thus, complete suppression of NF-Y expression may likely result in suppression of cell proliferation and cell death. Indeed, a knockout mouse for NF-YA is embryonic lethal, and embryonic fibroblasts prepared from this mouse demonstrate halted cell proliferation and DNA synthesis [35].

An important factor controlling tissue-specific expression of genes is CpG methylation [28]. This may particularly play a role in suppressing expression of NF-Y-responsive genes in many tissues since NF-Y is ubiquitously expressed [20-22] and is responsible for recruitment of RNA polymerase II [36]. However, the possibility exists that NF-Y may cooperate with another transcription factor(s), which could render expression of genes tissue and/or stage-specific. For instance, NF-Y and C/EBPα interact with each other and synergistically activate the mouse amelogenin gene, which contributes to physiological regulation during amelogenesis [37]. Whether this could be the case in the regulation of mouse *Scgb3a1 *gene expression remains to be determined.

DNA methylation is known to suppress gene expression through two basic mechanisms [38,39]; first, methylation of cytosine bases inhibit association between DNA-binding factors and their cognate DNA recognition sequences, resulting in direct inhibition of transcriptional activation. Second, proteins that recognize methyl-CpG such as methyl-CpG-binding proteins elicit repressive potential of methylated DNA by recruiting co-repressor molecules to silence gene transcription and to modify surrounding chromatin, including histone modification. In the case of the mouse *Scgb3a1 *gene, the second mechanism most likely plays a role in silencing expression of the gene since two CpGs are located closest to the transcription start site, which is 3–400 bp downstream of the NF-Y binding sites. These two CpGs in the mouse *Scgb3a1 *gene promoter are partially or completely unmethylated in mouse lung and mtCC cells, respectively. The partial methylation observed in the lung is the result of a mixture of methylated and unmethylated CpGs at approximately a 1:2 ratio. Although we do not have a proof, the methylated CpGs might be at least partially derived from non-epithelial cells such as mesenchymal cells that are contained in whole lung from which genomic DNA for methylation analysis was prepared. We found that the two CpGs were totally methylated when embryonic lung mesenchymal cells that do not express SCGB3A1 were separately prepared from epithelial cells and subjected to sequencing (Tomita and Kimura, unpublished results). SCGB3A1 is known to be expressed only in the airway epithelium [5]. However, very low SCGB3A1 expression was observed in the heart even though the two CpGs closest to the transcription start site were methylated. The reason for this difference is not known. A small percentage of these CpGs might be unmethylated in a specific part of the heart and not abundant enough to be detected particularly using mRNA prepared from whole heart. In this regard, the expression site in the heart is not known. Alternatively, another transcription factor(s) enriched in the heart might be responsible for the low SCGB3A1 expression in this tissue. EMSA using methylated CpG oligonucleotides revealed the presence of additional shifted bands as compared to unmethylated oligonucleotides. This could be due to binding of one of the methyl-CpG-binding proteins [38,40] or other DNA-binding proteins (transcription factors or co-factors) to these sites. We do not know which methyl-CpG-binding protein or DNA-binding protein is responsible for the new band, however the results at least suggest that CpG methylation affects binding of DNA-binding proteins within the region around proximal and distal CpGs in the mouse *Scgb3a1 *gene promoter. Previously we reported that a STAT binding element located at -201- to -209 bp of the mouse *Scgb3a1 *gene promoter is responsible for the IL-3/14-induced increase of *Scgb3a1 *gene expression in mtCC and mouse embryo lung primary cells [11]. This STAT element is located to close to the 3' most methylated CpG. Whether any changes in methylation status in this CpG under IL-3/14 induction remains to be determined.

Lastly, in the human *SCGB3A1 *gene promoter, 138 potential methylation CpG sites are found within 1,500 bp of the promoter region [1]. High frequency of methylation of these CpGs is associated with loss of SCGB3A1 expression in many human cancers [6-8]. In contrast, there are only 9 CpGs within 600 bp of the mouse *Scgb3a1 *gene promoter. This remarkable species difference in CpG islands can be explained by little significant homology in DNA sequences between the human and mouse gene promoters, suggesting that mouse *Scgb3a1 *and human *SCGB3A1 *may be under different regulation. This could explain the different patterns of SCGB3A1 expression between these two species. Whether the difference in gene regulation has any implication in functional role of SCGB3A1 between mouse and human requires additional studies.

## Conclusion

A ubiquitous transcription factor, NF-Y appears to be a potential potent regulator of mouse *Scgb3a1 *gene expression through binding to two "CCAAT" boxes located at -425 to -429, and -498 to -502 bp in the *Scgb3a1 *gene promoter. Tissue-specific expression of SCGB3A1 may be due in part to methylation of the two CpG sites closest to the transcription start site of the gene.

## Methods

### Materials

Normal rabbit IgG (sc-2027), anti-NF-YA (sc-10779X) and anti-NF-YB (sc-13045) antibodies were purchased from Santa Cruz Biotechnology (Santa Cruz, CA). All restriction endonucleases were obtained from New England Biolabs (Ipswich, MA).

### Plasmid construction

Serial *Scgb3a1 *gene promoter deletion mutants were constructed with PCR using mouse genomic DNA and the following primers: forward primer for -59 construct 5'-CAAACTAGTTCCCAGGAGGGTGAGGTTCCAC-3', -91 construct 5'-TAAGCTAGCCCTTTCTGCCTGTTAGCTGAGCAAAC-3', -184 construct 5'-CACACTAGTTCAGGAGACCCTGCCAAGAG-3', -273 construct 5'-AGGACTAGTCAGACCACATTATCAAGGTGTAGG-3', and -598 construct 5'-CCCACTAGTCATTAGCAGCTACCTGTGGCCCAAC-3', and a common reverse primer 5'-AGAGGATCCCAGGACCTATAAGACAATCTTCC-3'. PCR products were double-digested with Spe I (Nhe I for -91 construct) and Bam HI, and cloned into the Nhe I – Bgl II site of the pGL4.11 vector (Promega, Madison, WI). These reporter constructs contained +24 bp of the 5' UTR of the SCGB3A1 mRNA sequence.

For construction of expression plasmids, total RNAs isolated from mouse embryonic lungs (embryonic day 16.5) by using TRIZOL (Invitrogen, Carlsbad, CA) were first treated with DNase I (Ambion, Austin, TX) and subjected to cDNA synthesis using Superscript II reverse transcriptase (Invitrogen). PCR reactions were carried out using the following primers: 5'-TTAGCGGCCGCATGGAGCAGTATACGACAAACAGCAATAG-3' and 5'-TAATCTAGATTAGGAAACTCGGATGATCTGTGTCATGG-3' for NF-YA, 5'-TAAGGTACCTTACATGACAATGGACGGCGACAGCTC-3' and 5'-TAATCTAGATCATGAAAACTGAATTTGCTGGACACCAG-3' for NF-YB, 5'-TAAGCGGCCGCACCATGTCCACAGAAGGAGGGTTTGG-3' and 5'-TTATCTAGAGCCCTCAGTCTCCAGTCACCTGG-3' for NF-YC, and 5'-TTAGCGGCCGCATGTTACAGGCGTGCAAAATGGAAGG-3' and 5'-TTATCTAGACGATCAGTGGGGCGGGAGG-3' for PU.1/Spi-1. PCR products were subcloned into the Not I – Xba I (NF-YA, NF-YC and Spi-1) or Kpn I – Xba I sites (NF-YB) of pcDNA3.1 vector (Invitrogen). The NF-Y binding site and PU-box mutations were generated by site-directed mutagenesis kit (Stratagene, La Jolla, CA). All plasmid sequences were confirmed by DNA sequence analyses (Beckman Coulter, model CEQ-200XL, Fullerton, CA).

### Reporter gene assays

COS-1 and mtCC cells were seeded in 24-well tissue culture plates in DMEM with high glucose and L-glutamine (Invitrogen), supplemented with 10% FBS. A transfection cocktail contained 20 μl serum-free DMEM, 1 μl Fugene 6 (Roche Applied Science, Indianapolis, IN), 250 ng reporter construct, and 5 ng pGL4.74 Renilla luciferase vector (Promega) as an internal control, and whenever necessary, a various combination of three NF-Y expression plasmids (16.6 ng each, a total amount adjusted to 50 ng with a control vector). In order to have all plasmids transfected into a cell, the amount of each expression plasmid used were kept small and transfection was carried out using cells at 50% confluency. Cells were washed with PBS 48 h after transfection, and lysed in passive lysis buffer (Promega). Luciferase activity was determined using a luminometer (Pharmingen, model monolight 3010) with Dual-Luciferase Reporter Assay System (Promega). All reporter assays were carried out at least three times, each in duplicate, and the results were expressed as the mean ± SD.

For shRNA experiments, synthetic oligonucleotide designed to generate RNAi for mouse NF-YA (Invitrogen, Mmi515428) was inserted into a linearized vector pcDNA6.2-GW/miR (Invitrogen). mtCC cells transfected with Mmi515428/pcDNA6.2-GW/miR or control pcDNA6.2-GW/miR were maintained in media containing 2 μg/ml blasticidin (Invitrogen).

### Nuclear extracts

Nuclear extracts were prepared from COS-1 cells transfected with NF-Y expression plasmids, mouse lungs and mtCC cells [41]. In the case of COS-1 cells, 1 ml of serum-free DMEM containing expression constructs (NF-YA, NF-YB, and NF-YC, 7 μg each) was mixed with 50 μl of Fugene HD (Roche Applied Science, Indianapolis, IN), which after 15 min incubation, added to COS-1 cells that were grown to 50–80% confluency in 150-mm dish. The media was changed 8 h after transfection, and 48 h after the media change, cells were washed and harvested in cold PBS. Cell suspension was centrifuged for 5 min at 1,000 rpm and the pellet was resuspended in 2 ml Buffer A (10 mM HEPES, pH 7.6, 15 mM KCl, 2 mM MgCl2, 0.1 mM EDTA, 1 mM DTT, 0.5 mM PMSF). After centrifugation at 2,500 rpm for 3 min, cell pellet was suspended in Buffer B (Buffer A + 0.2% IGEPAL), followed by re-centrifugation for 3 min at 2,500 rpm. Mouse lungs were homogenized using a dounce homogenizer in Sucrose buffer (250 mM sucrose, 10 mM HEPES, pH 7.6, 15 mM KCl, 2 mM MgCl2, 0.5 M EDTA, 1 mM DTT, 0.5 mM PMSF), and centrifuged for 3 min at 2,500 rpm. The nuclear pellets thus obtained were washed with Sucrose buffer mixed with Extraction buffer (50 mM HEPES, pH 7.9, 400 mM KCl, 0.1 mM EDTA, 10% Glycerol, 0.5 mM PMSF), and rotated in cold room for 30 min. Finally, the nuclear extract was obtained by centrifugation, and protein concentration was adjusted at 2 mg/ml by using Bradford protein assay (Bio-Rad Laboratories, Hercules, CA) with BSA as a standard.

### Electrophoretic mobility shift assays (EMSA)

Oligonucleotides were radio-labeled with [γ-^32^P]ATP (PerkinElmer, Wellesley, MA) and T4 polynucleotide kinase (New England Biolabs). Nuclear extract (1 μl) was diluted in Binding buffer (0.1 μg/μl polydI-dC, 10 mM Tris, pH 8.0, 1 mM DTT, 80 mM KCl, 20% Glycerol, 0.04 μg/μl BSA) and incubated for 15 min at room temperature with radio labeled probe in the presence or absence of cold oligonucleotide as a competitor. For supershift analysis, the mixture was incubated with 1 μl of antibody solution for additional 15 min at room temperature. DNA-protein complexes were electrophoresed on a 4% polyacrylamide gel using 0.5× TBE buffer, and were visualized by exposing to phosphoimager screen (Storm 840, Amersham Biosciences, Piscataway, NJ).

### Chromatin immunoprecipitation (ChIP) assays

DNA was isolated from mtCC, NIH3T3, and MLE15 cells that were fixed with 1% formaldehyde, followed by sonication. MLE15 was grown in HITES (RPMI-1640 supplemented with insulin, transferrin, sodium selenite [Sigma, St. Louis, MO], 10 mM hydrocortisone [Sigma], 10 mM γ-estradiol [Sigma], 10 mM HEPES [Invitrogen], and 2%FCS). Endogenous NF-Y together with fragmented DNAs was pulled down by anti-NF-YA or rabbit IgG as control. After reverse cross-linking, the *Scgb3a1 *promoter region containing the NF-Y binding site (200 bp) and a region derived from *Scgb3a2 *intron 1 + exon 2 as a reference (219 bp) were PCR amplified. PCR primers used were as follows: 5'-GCTATTAGGCATTCCTTTCTGGCTC-3' and 5'-CCACGGGCGATTATGTAGGTTC-3' for amplification of the NF-Y binding site, and 5'-TCTTCAGTCCTGTCACCAGATGTTCTAC-3' and 5'-CGAGAGGGATGGGATGGAGTCTTAG-3' for the reference. Sample solution was mixed with each primer set and *Taq *polymerase, followed by PCR reaction of 25 cycles with 94°C denaturation, 15 sec, 56.5°C annealing, 15 sec, and 72°C extension, 30 sec.

### RT-PCR analysis

RNAs isolated from various organs of adult mouse and various mouse cell lines were subjected to RT-PCR analysis. PCR was carried out after cDNA synthesis using the following primers: 5'-CTCTACAGATCCCAGGCAGC-3' and 5'-CTGGAGCCTCTGATTGGGT-3' for NF-YA, 5'-TAGCTGGGAGGCATCTGTG-3' and 5'-AGGATCCACCACCTTTTTGA-3' for NF-YB, 5'-TTTCTTCCATGACTCTGGGC-3' and 5'-GCTGCTTTCTTCGCTGGA-3' for NF-YC, and 5'-GATGGCCAAGTGGCTTAATG-3' and 5'-TCTGTGTGGCTCTGCTCAGT-3' for SCGB3A1. PCR condition used was 94°C, 2 min, followed by 30 cycles of 94°C, 15 sec, 55°C, 15 sec, and 68°C, 30 sec.

### Northern blot

Total RNA (3 μg) isolated from mtCC cells was electrophoresed on 1% agarose gel containing 0.22 M formaldehyde and transferred onto nitrocellulose membrane (Immobilon-Ny+, Millipore, Billerica, MA). Filters were hybridized with mouse SCGB3A1 and ribosomal protein B36 (loading control) as a probe. Hybridization was performed in Perfect Hybridization solution (Amersham Biosciences) at 68°C overnight. The membrane was washed twice with 2 × SSC containing 0.1% SDS at 68°C for 30 min, followed by exposure to phosphoimager screen. Data processing was carried out using ImageQuant TL 2005 software (Amersham Biosciences).

### Western blot

Nuclear extracts (3 μg) obtained from mtCC cells were run on 10% SDS-polyacrylamide gels, and proteins transferred onto PVDF membrane (Hydond-P, Amersham Biosciences). The membrane was gently shaken with PBS containing 5% skim milk at 4°C overnight, followed by incubation with anti-NF-YA or NF-YB antibody diluted in PBS containing 1% Tween 20 (PBST). After washing three times with PBST, the membrane was incubated with horseradish peroxidase conjugated anti-rabbit IgG (NA9340V, Amersham Biosciences) followed by further three times wash with PBST. Signals were directly detected and quantified using chemiluminescence reaction (Immobilon Western, Millipore) with CCD camera system and equipped software (Alpha Innotech Fluor Chem HD2, San Leandro, CA).

### DNA methylation analysis

Genomic DNAs were isolated based on simplified procedure [42] from various tissues and cultured cells. Isolated DNAs were overnight digested with Bam HI at 37°C, ethanol precipitated after phenol-chloroform extraction and pellets were dissolved in TE buffer. EZ DNA methylation kit (Zymo Research, CA) was used to generate bisulfite treated DNA. Five hundred ng of DNA was incubated with bisulfite at 50°C for 16 h, and the product was purified and desulphonated through a spin-column. This procedure converts all non-methylated cytosine to uracil but does not affect 5-methyl cytosine. Two μl of final elution from the spin-column was used as template for two different PCRs with a primer set, 5'-GTGGTTTTTGGAAGAAAGGTTTAGTATTTGAGTTTAGG-3' and TACTAAACCCCCAAAAAAACTCACCAAAAATCAC-3' for proximal *Scgb3a1 *promoter region (343 bp), and 5'-GAGAGATTTAGAGTTTTTGGGATTTGTTGATTTAT-3' and 5'-CCCACCTCAATCCTAAAAATTTCCTCTTAAC-3' for distal *Scgb3a1 *promoter region (279 bp). Both PCR reactions were carried out at the same conditions; 94°C, 2 min, followed by 40 cycles of 94°C, 15 sec, 52.8°C, 15 sec, and 68°C, 30 sec. All PCR products were subjected to 2% agarose gel electrophoresis, and each band was excised and purified (gel extraction kit, Qiagen, Valencia, CA) for subsequent direct sequencing analysis to identify cytosine residues, but not thymine (uracil), that was the result of resistance to bisulfite reaction due to methylation. In some cases, PCR products were subcloned into pGEM-T easy vector (Promega) and individual clones were subjected to sequencing analysis. For confirmation of non-bisulfite treated genomic DNA sequence, PCR was carried out using the following primer sets: 5'-CAGAAAAATGTCACAGCCCCTC-3' and 5'-CCAACTTCCTCTTATGGTCAGTGGAC-3' to obtain 543-bp fragment of the distal *Scgb3a1 *promoter region containing the two CCAAT sites and 5'-CTTCCAGACCCAGAACCTACATAATC-3' and 5'-AGAGTCACTGAGCAGAGCCACAC-3' to obtain 471-bp fragment of the proximal *Scgb3a1 *promoter region. PCR condition used was 94°C, 2 min, followed by 30 cycles of 94°C, 15 sec, 58°C, 15 sec, and 68°C, 60 sec.

## Authors' contributions

TT performed all experiments and drafted the manuscript. SK coordinated this study, provided its conceptual basis, participated in experimental design and helped to draft the manuscript. Both authors read and approved the final manuscript.
